# A machine learning framework that integrates multi-omics data predicts cancer-related LncRNAs

**DOI:** 10.1186/s12859-021-04256-8

**Published:** 2021-06-16

**Authors:** Lin Yuan, Jing Zhao, Tao Sun, Zhen Shen

**Affiliations:** 1 School of Computer Science and Technology, Qilu University of Technology (Shandong Academy of Sciences), Daxue Road 3501, Jinan, 250353 Shandong China; 2 School of Computer and Software, Nanyang Institute of Technology, Changjiang Road 80, Nanyang, 473004 Henan China

**Keywords:** LncRNA, Multi-omics data, Machine learning, Neural network, Node embedding, Cancer

## Abstract

**Background:**

LncRNAs (Long non-coding RNAs) are a type of non-coding RNA molecule with transcript length longer than 200 nucleotides. LncRNA has been novel candidate biomarkers in cancer diagnosis and prognosis. However, it is difficult to discover the true association mechanism between lncRNAs and complex diseases. The unprecedented enrichment of multi-omics data and the rapid development of machine learning technology provide us with the opportunity to design a machine learning framework to study the relationship between lncRNAs and complex diseases.

**Results:**

In this article, we proposed a new machine learning approach, namely LGDLDA (LncRNA-Gene-Disease association networks based LncRNA-Disease Association prediction), for disease-related lncRNAs association prediction based multi-omics data, machine learning methods and neural network neighborhood information aggregation. Firstly, LGDLDA calculates the similarity matrix of lncRNA, gene and disease respectively, and it calculates the similarity between lncRNAs through the lncRNA expression profile matrix, lncRNA-miRNA interaction matrix and lncRNA-protein interaction matrix. We obtain gene similarity matrix by calculating the lncRNA-gene association matrix and the gene-disease association matrix, and we obtain disease similarity matrix by calculating the disease ontology, the disease-miRNA association matrix, and Gaussian interaction profile kernel similarity. Secondly, LGDLDA integrates the neighborhood information in similarity matrices by using nonlinear feature learning of neural network. Thirdly, LGDLDA uses embedded node representations to approximate the observed matrices. Finally, LGDLDA ranks candidate lncRNA-disease pairs and then selects potential disease-related lncRNAs.

**Conclusions:**

Compared with lncRNA-disease prediction methods, our proposed method takes into account more critical information and obtains the performance improvement cancer-related lncRNA predictions. Randomly split data experiment results show that the stability of LGDLDA is better than IDHI-MIRW, NCPLDA, LncDisAP and NCPHLDA. The results on different simulation data sets show that LGDLDA can accurately and effectively predict the disease-related lncRNAs. Furthermore, we applied the method to three real cancer data including gastric cancer, colorectal cancer and breast cancer to predict potential cancer-related lncRNAs.

**Supplementary Information:**

The online version contains supplementary material available at 10.1186/s12859-021-04256-8.

## Background

Long non-coding RNAs (lncRNAs) are a type of non-coding RNA molecule with transcript length longer than 200 nucleotides [1, 2]. Many studies have confirmed that the human genome contains massive amounts of lncRNA [3]. Many evidences indicate that lncRNAs regulate the expression level of genes at multiple levels (e.g., epigenetic regulation, genomic splicing, genomic imprinting, chromatin modification, transcriptional activation, transcriptional and post-transcriptional regulation) in the form of RNA [4–7]. The aberrant expression of lncRNA is involved in the proliferation, apoptosis, angiogenesis, and metastasis of tumors [8, 9]. LncRNA is closely related to the diagnosis, prognosis, and prevention and treatment of complex diseases [10]. LncRNA has become a new candidate biomarker for cancer diagnosis and prognosis [11].

The experimentally verified information about disease-related lncRNA is gradually increasing. A large number of databases have been published. The database LncRNADisease contains 3000 lncRNA-disease associations [12]. The database Lnc2Cancer has collected 1500 lncRNA-cancer entries [13]. Moreover, researchers have constructed lncRNA-related databases including NONCODE [14], lncRNAdb [15], LNCipedia [16], lncACTdb [17]. Although the research on lncRNA has progressed rapidly in recent years, the functions of most lncRNAs are still unclear. Bioinformatics calculation methods have been developed to predict the potential lncRNA-disease associations for biological experiment verifications. The calculation methods can greatly reduce the experimental cost and time for finding new disease-related lncRNAs [18, 19].

The disease-related lncRNAs prediction methods can be categorized into network-based approaches and machine learning-based approaches. Biological system is a highly complex heterogenous network involving different molecules. Network-based approaches use multiple features including (but not limited to) lncRNA functional similarity, lncRNA-gene association, gene–gene interaction, gene-disease association, and molecular similarity to construct lncRNA similarity networks, or lncRNA-disease heterogeneous networks, then use network model analysis methods (e.g. propagation algorithms and random walk theory) to predict potential lncRNA-disease associations [20]. RWRlncD constructed a unified network including disease similarity network, lncRNA functional similarity network, and disease-lncRNA association network. The method used the Random Walk with Restart (RWR) method to predict the potential lncRNA-disease association [21]. RWRHLD added miRNA information that interacts with lncRNA, further improving the accuracy of the lncRNA-disease prediction method [22]. LncRDNetFlow used a streaming algorithm to predict lncRNA-disease associations based on multi-omics networks [23]. However, the known lncRNA-disease association data is still insufficient, and those methods cannot be applied to the prediction of related disease without any known lncRNAs information. To avoid the abovementioned problems, researchers attempt to combine known pathogenic gene-miRNA association data, miRNA-lncRNA association data and other data to predict lncRNA-disease association. LncPriCNet used multiple features, including phenotype-gene relations and gene–gene interactions, to construct a multi-level composite network and then used similarity scores to predict lncRNA-disease associations [24]. Ganegoda et al. proposed a model for predicting potential disease-associated lncRNAs by integrating known cancer-associated lncRNAs information and multi-omics data including genomic, regulatory, and transcriptional bios data [25].

Recently, many bioinformatics calculation models based on machine learning algorithms have been proposed to find potential lncRNA-disease associations. Lu et al. used inductive matrix completion and principal component analysis to predict potential lncRNA-disease associations [26]. Based on a review of existing research, Chen et al. proposed a hypothesis that functionally similar lncRNAs tend to be abnormally expressed in similar diseases, and developed a semi-supervised machine learning framework based on laplacian regularized least squares method (named LRLSLDA). Unfortunately, the method suffered from selecting multiple parameters effectively [27]. Wang et al. used lncRNA similarity data and disease similarity data to train a bagging support vector machine (SVM) classifier, and the trained SVM is implemented as a web server to predict potential disease-related lncRNAs [28]. You et al. proposed a method called LDASR to predict latent lncRNA-disease associations by using collaborative filtering and rotating forest [29]. These methods have achieved good results. Although the research on lncRNA has made rapid progress in recent years, unfortunately, these methods often used unmodified traditional machine learning methods, and the omics data used are limited to two or three types. Recently, the accumulation of associated omics data between lncRNAs and diseases and the development of machine learning and deep learning technologies provide researchers with better opportunities to use supervised learning models to predict disease-related lncRNAs.

Meanwhile, modern medical research proves that the alternations of biological factors (e.g., miRNA, protein and gene) may directly or indirectly affect diseases. Earlier studies have shown that RNA–protein interactions regulate gene expression by controlling various post-transcriptional processes. LncRNAs regulate the RNA–protein interactions by recruiting regulatory complexes [30, 31], and the literatures indicate that many lncRNAs also act as regulators to regulate gene expression [32]. Wang et al. reported that lncRNA-miRNA-disease interactive network could be great addition to the biomedical research field [33]. Liu et al. reported that lncRNA-binding proteins play a key role in the development of many diseases [34]. The accumulated miRNA-disease associations can be used for disease treatment [35]. Considering the mechanism of lncRNAs regulate genes, and biological factors regulate diseases provide a better opportunity for obtaining more information about lncRNA-disease associations.

Inspired by currently well-performing neural network technologies [36, 37], we tried to use multiple omics similarity matrices, neural network neighborhood information aggregation and trained supervised learning model to extract association features from lncRNA-gene-disease association network to predict disease-related lncRNAs. In this article, we proposed a new machine learning framework named LGDLDA (LncRNA-Gene-Disease association networks based LncRNA-Disease Association prediction) for disease-related lncRNAs association prediction based multi-omics functional similarity data, machine learning methods and neural network neighborhood information aggregation. We collected data from three databases LncRNADisease v2.0 [38], Lnc2Cancer [13], and MNDR v2.0 databases [39] separately, and then combined these three data into one data. The diseases in this combined data do not include gastric cancer, breast cancer, and prostate cancer. Additional file 1: Fig. S1 provided the data processing procedure for disease-lncRNA association instances. This combined data contains 6000 disease-lncRNA association instances, of which 4000 association instances were used for training and 2000 association instances were used for validating. Firstly, LGDLDA calculates the similarity between lncRNAs through the lncRNA expression profile matrix, lncRNA-miRNA interaction matrix and lncRNA-protein interaction matrix. The gene similarity matrix is obtained by calculating the lncRNA-gene association.

matrix and the gene-disease association matrix. The disease similarity matrix is obtained by calculating the disease ontology, the disease-miRNA association matrix, and Gaussian interaction profile kernel similarity. Secondly, LGDLDA integrates neighborhood information by using nonlinear feature learning of neural network. Thirdly, LGDLDA uses embedded node representations to approximate the observed matrices. Finally, LGDLDA ranks candidate lncRNA-disease pairs and then selects potential disease-related lncRNAs. The stability test results show that LGDLDA is more robust and the simulation data experiments show that LGDLDA performs better than four state-of-art methods in predicting lncRNA-disease association. LGDLDA can effectively predict potential cancer-related lncRNAs and provide more candidates for biological experimental verification. Most of predicted cancer-related lncRNAs are supported by recent literatures.

## Results

In the results section, the work we do is described as follows: Firstly, we used randomly split samples to observe the robustness of each method. Secondly, we compared LGDLDA with four famous lncRNA-disease association prediction methods on a small lncRNA-disease association simulation network. Four state-of-art methods include NCPLDA [40], IDHI-MIRW [41], LncDisAP [42] and NCPHLDA [43]. Finally, LGDLDA was applied to three real cancer samples to predict potential disease-related lncRNAs.

### Comparison of method stability

Before comparing the performance of LGDLDA with four famous lncRNA-disease association prediction methods in small data, we need to evaluate the stability of these methods. We generally randomly divide the data set into two parts: Ω1 and Ω2. In the first step, based on the training set Ω1, we select different parameters and determine the parameter configuration with good performance. In the second step, we expect that the selected parameter configuration can have an accurate prediction in Ω2. We performed this experiment on a small lncRNA-disease association simulation network which contains 356 lncRNAs, 354 diseases, 132 genes, 736 known lncRNA-gene associations, 462 gene-disease associations and 2169 known lncRNA-disease association instances [41]. Ω1 contains 1446 lncRNA-gene association instances and Ω2 contains 723 lncRNA-gene association instances. There may be two issues to consider: (i) Does the randomness in the randomly divided sample affect the stability of the method? (ii) Is the stability of LGDLDA better than NCPLDA [40], IDHI-MIRW [41], LncDisAP [42] and NCPHLDA [43] ?

**Fig. 1:**
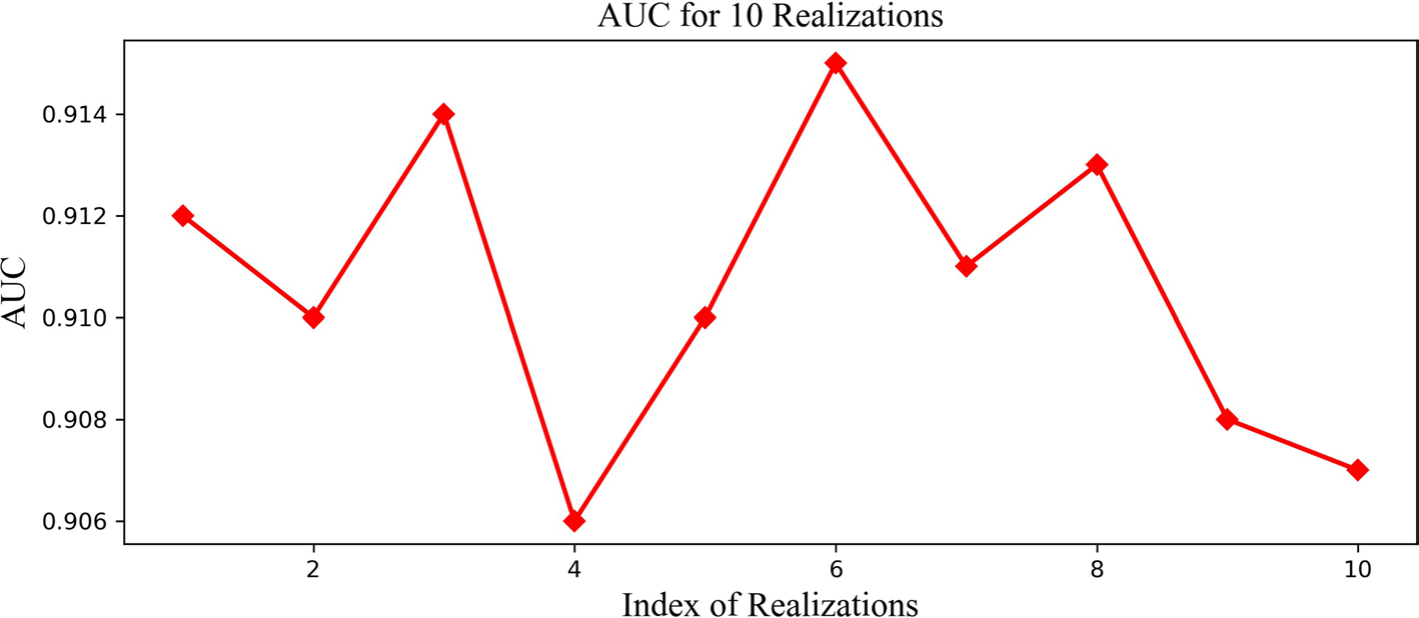
The AUC values for 10 realizations

**Fig. 2:**
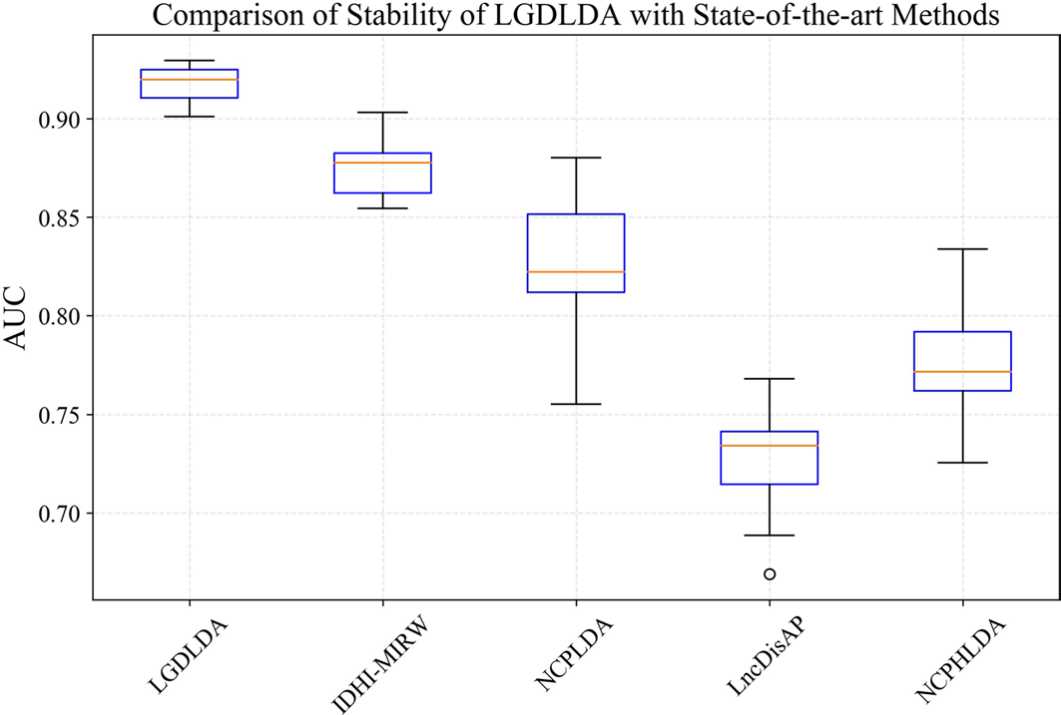
The box plots of LGDLDA, IDHI-MIRW, NCPLDA, LncDisAP and NCPHLDA

To address the two issues, we observed the performance of the method in two experiments. In the first experiment, we performed 10 random splits on a certain comprehensive data set. For each randomly divided data set, we ran LGDLDA on the data set and calculated AUC values. The AUC values for 10 realizations are shown in Fig. 1. The experimental results from Fig. 1 show that random partition strategy has little effect on the method performance. In the second experiment, we performed 50 random splits on a certain comprehensive data set. For each randomly divided data set, we ran each method on the data set and calculated AUC values. BasedWe performed these experiments on these AUC values, we calculated the minimum, first quartile, median, third quartile and maximum value and draw boxplots. The box plots from Fig. 2 show that the stability of LGDLDA is better than IDHI-MIRW, NCPLDA, LncDisAP and NCPHLDA. We also performed 10 random splits experiment and 50 random splits experiment on a dataset with 10% incorrect data. The AUC values for 10 realizations on the dataset are shown in Additional file 1: Fig. S2. The box plots from 50 random splits experiment on a dataset with 10% incorrect data are shown in Additional file 1: Fig. S3.

### Comparison with four state-of-art methods on a small simulation data set

In this section, we compared LGDLDA with four famous methods (i.e., NCPLDA, IDHI-MIRW, LncDisAP and NCPHLDA) on a small lncRNA-disease association simulation network which contains 356 lncRNAs, 354 diseases, 132 genes, 736 known lncRNA-gene associations, 462 gene-disease associations and 2169 known lncRNA-disease associations from breast cancer [41]. LncDisAP [42] and IDHI-MIRW [41] are prediction methods based on multiple biological datasets and RWR algorithm. NCPHLDA [43] and NCPLDA [40] are network-based methods. We performed these experiments on a computer with an Intel i9-10900X CPU and 512 G RAM.

**Fig. 3:**
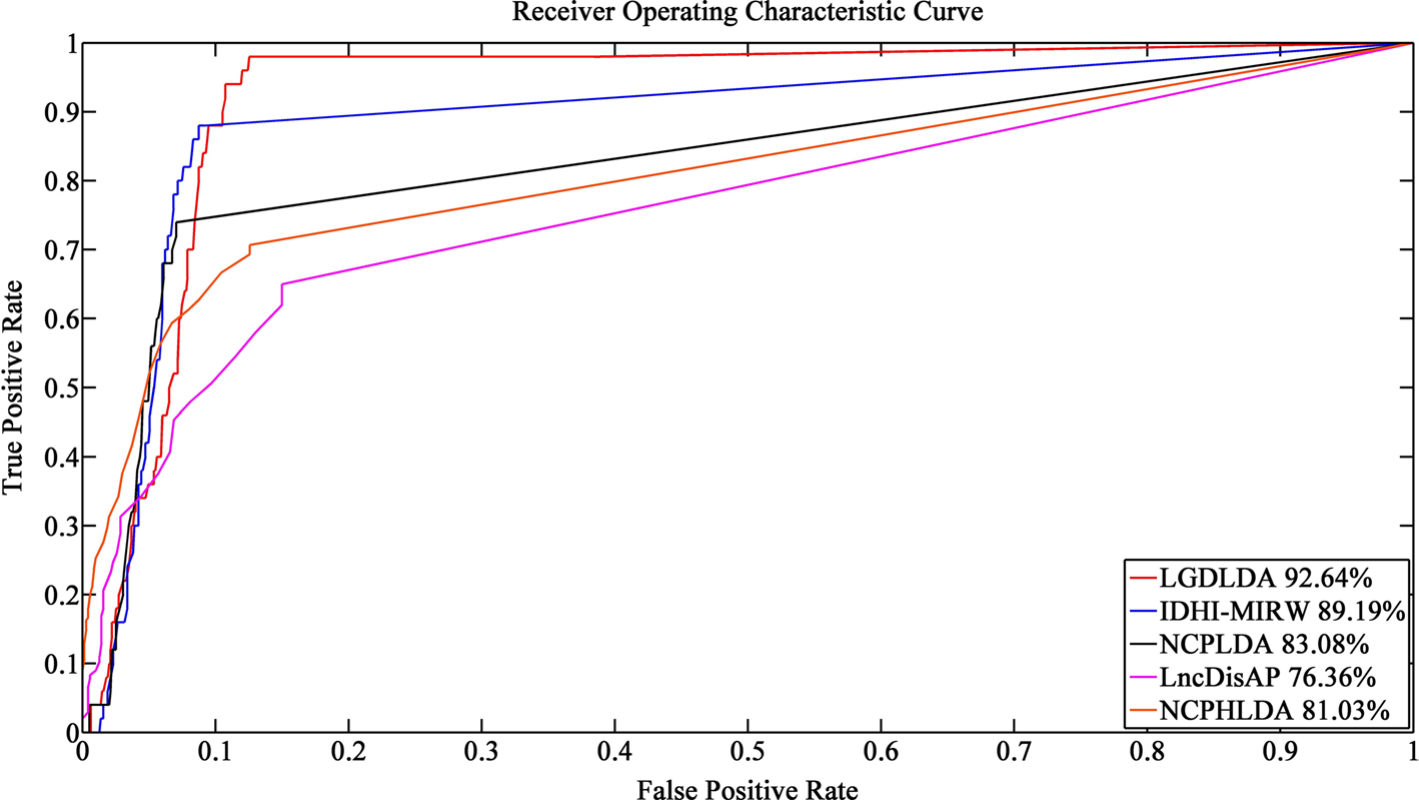
The ROCs and corresponding AUC values of LGDLDA, IDHI-MIRW, NCPLDA, LncDisAP and NCPHLDA on data that does not contain gene-related information

**Fig. 4:**
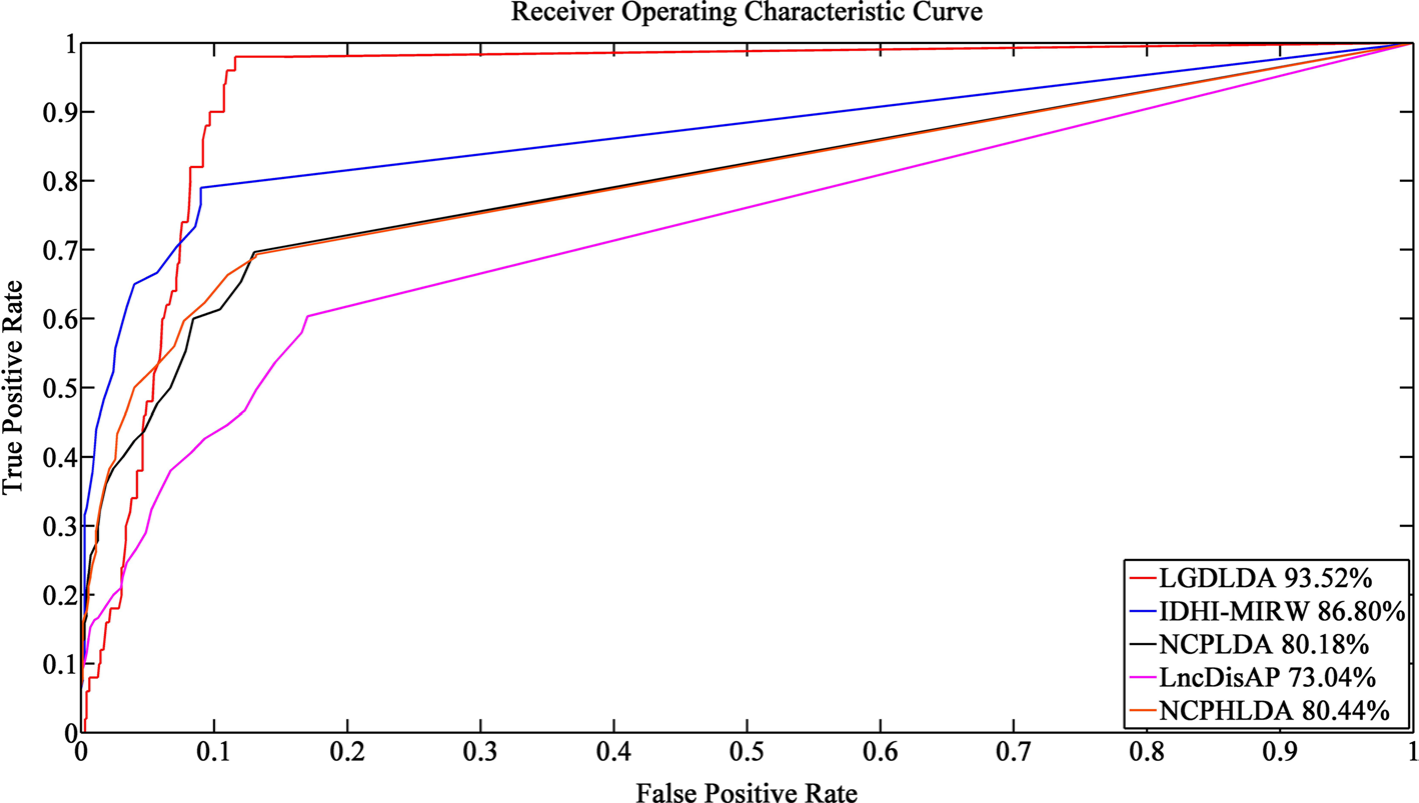
The ROCs and corresponding AUC values of LGDLDA, IDHI-MIRW, NCPLDA, LncDisAP and NCPHLDA on data containing gene information

**Fig. 5:**
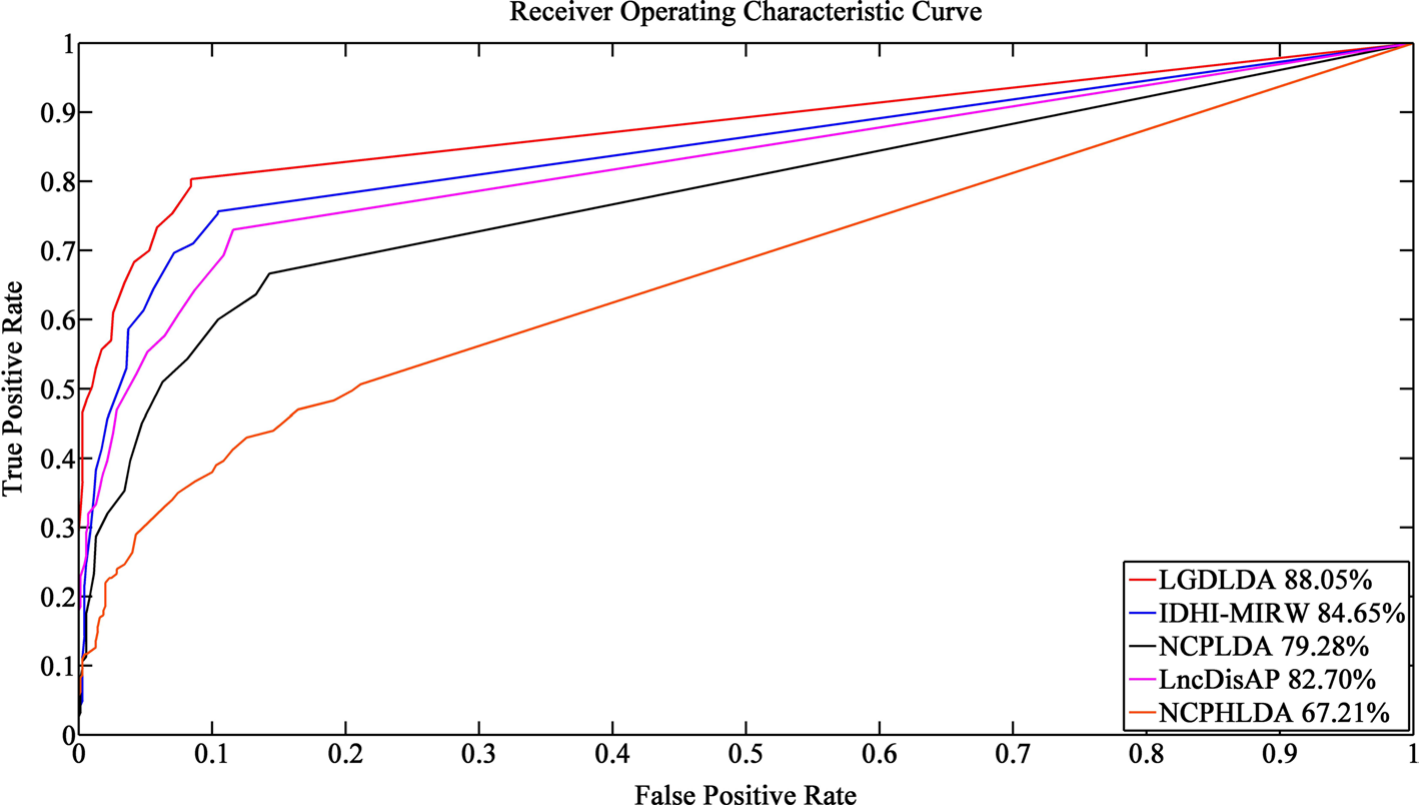
The ROCs and corresponding AUC values of LGDLDA, IDHI-MIRW, NCPLDA, LncDisAP and NCPHLDA on the data with missing part of the information

To avoid the small lncRNA-disease association simulation network favoring our own model, we run each method on data that does not contain gene-related information (i.e., data without genes, lncRNA-gene associations, and gene-disease associations). Figure 3 shows the ROCs and corresponding AUC values of LGDLDA and four competition methods. As shown in Fig. 3, LGDLDA outperformed other four methods in terms of AUC value. The AUC of LGDLDA is 0.926, which is 0.035, 0.096, 0.163 and 0.116 higher than that of IDHI-MIRW, NCPLDA, LncDisAP and NCPHLDA, respectively. We also run each method on data containing gene information. Figure 4 shows the ROCs and AUC values of LGDLDA and the four competition methods. As shown in Fig. 4, LGDLDA outperformed other four methods in terms of AUC value. The AUC of LGDLDA is 0.935, which is 0.067, 0.134, 0.205 and 0.131 higher than that of IDHI-MIRW, NCPLDA, LncDisAP and NCPHLDA, respectively. Considering we often apply method to incomplete data set, we randomly remove 20% of the data and run each method. The ROCs and AUC values of LGDLDA and other four methods are shown in Fig. 5. LGDLDA achieved a better performance than other four methods in terms of AUC. The AUC of LGDLDA is 0.880, which is 0.034, 0.088, 0.053 and 0.208 higher than that of IDHI-MIRW, NCPLDA, LncDisAP and NCPHLDA, respectively. Although our method LGDLDA is affected by incomplete data, it performs better than other four methods. Compared with the four state-of-art methods, the results on different simulation data sets show that LGDLDA can accurately and effectively predict the disease-related lncRNAs.

In order to observe whether it is necessary to include each omics data, we performed the experiment on the dataset with missing part of the omics data and recorded the AUC values, and compared with the experimental results on the complete multi-omics dataset. The experimental results are shown in Additional file 1: Table S1.

### Application to cancer data and potential lncRNA-disease associations analysis

In this section, we applied LGDLDA to real cancer data including gastric cancer, colorectal cancer, and breast cancer. For a given disease, all known related lncRNAs are true labels, and other lncRNAs are candidates for disease. Inspired by the work of Guo et al. [29], we used the related information in the LncRNADisease database v2.0, DisGeNet, and LncACTdb to train LGDLDA, and other databases including CRlncRNA [44], MNDR v2.0, LncRNAwiki [45], and Lnc2Cancer, were used to verify the results. We applied the LGDLDA to real cancer data and ranked the lncRNA-disease association scores from large to small, and then identified the top 15 potentially relevant lncRNAs for each cancer.

Gastric cancer is the second most common cancer in the world [46, 47]. Accumulating evidence has demonstrated that many lncRNAs are dysregulated in gastric cancer [48, 49]. It is necessary to use computing methods to predict cancer-related lncRNAs. In the gastric cancer study, we used 1352 associations and gene related associations from databases as positive samples. We randomly selected the same number of samples from the database as negative samples. We constructed the test data set by extracting gastric cancer-related lncRNAs from other databases. Recent literatures supported 12 out of 15 potential gastric cancer-related lncRNAs. The confirmed databases and supporting literature of these 15 cancer-related lncRNAs are shown in Table 1 and Additional file 1: Table S2, respectively. For example, Xu et al. [50] found that overexpression of ZFAS1 is significantly related to lymphatic metastasis and TNM staging. The overexpression of ZFAS1 leads to the loss of control of the cell cycle process, which in turn promotes the proliferation and migration of gastric cancer cells. Liu et al. reported that lncRNA H19 is aberrantly highly expressed in gastric cancer cell lines. Zai et al. reported that activated DANCR promotes the proliferation and invasion of gastric cancer cells [51]. LncRNA HOXA11-AS promotes the invasion and proliferation of gastric cancer by regulating the chromatin modifiers LSD1 and DNMT1 [52]. A large number of studies have shown that LncRNA can be used as a biomarker for the treatment of gastric cancer [53].

**Table 1:** The confirmed databases of Top 15 gastric cancer-associated LncRNAs predicted by LGDLDA

Rank	Name of LncRNA	Confirmed database
1	UCA1	CRlncRNA/LncRNAWiki/Lnc2Cancer/LncRNADisease v2.0
2	NEHG1	Unconfirmed
3	TINCR	Lnc2Cancer/LncRNAWiki/LncRNADisease v2.0
4	HOTAIR	Lnc2Cancer/LncRNAWiki/LncRNADisease v2.0
5	C1RL-AS1	Unconfirmed
6	SPRY4-IT1	Lnc2Cancer/LncRNAWiki/LncRNADisease v2.0
7	PVT1	CRlncRNA/Lnc2Cancer/LncRNADisease v2.0
8	NEAT1	LncRNAWiki/LncRNADisease v2.0/CRlncRNA
9	MEG3	LncRNAWiki/Lnc2Cancer/LncRNADisease v2.0
10	MALAT1	LncRNAWiki/Lnc2Cancer/LncRNADisease v2.0
11	DM1-AS	Unconfirmed
12	MIAT	CRlncRNA/LncRNADisease v2.0
13	GHET1	LncRNAWiki/Lnc2Cancer/LncRNADisease v2.0
14	FER1L4	Lnc2Cancer/LncRNAWiki/LncRNADisease v2.0
15	SUMO1P3	Lnc2Cancer/LncRNAWiki/LncRNADisease v2.0

Breast cancer is the most common malignant tumor in women and the second leading cause of cancer death [54, 55]. If we can detect cancer-related lncRNA as early as possible and intervene early, it will greatly reduce the incidence of breast cancer. Recent literatures supported 12 out of 15 potential breast cancer-related lncRNAs. The confirmed databases and supporting literature of these 15 cancer-related lncRNAs are shown in Additional file 1: Table S3 and Additional file 1: Table S4, respectively. For example, Yang et al. found that overexpression of LncRNA BCRT1 can promote the M2 polarization of macrophages, thereby accelerating the development of breast cancer [56]. Schiemann reported that lncRNA BORG regulates the transcriptional repressive activity of TRIM28 to trigger the migration and invasion of potential breast cancer cells [57]. Spector et al. reported that lncRNA MaTAR25 affects the proliferation and metastasis of breast cancer cells by regulating the expression of Tensin1 gene [58].

Prostate cancer is the second most common cancer in men and the fifth leading cause of death worldwide [59, 60]. Recent literatures supported 12 out of 15 potential prostate cancer-related lncRNAs. The confirmed databases and supporting literature of these 15 cancer-related lncRNAs are shown in Additional file 1: Table S5 and Additional file 1: Table S6, respectively. For example, Zhao et al. [61] reported that overexpression of ANRIL promoted the proliferation and migration of prostate cancer cells. Li et al. reported that lncRNA SNHG1 enhanced the expression of CDK7 and promoted cell proliferation in prostate cancer by negatively regulating miR-199a-3p [62]. Zhang et al. reported that the androgen-reduced transcript of LncRNA GAS5 can promote the proliferation of prostate cancer [63].

## Discussion

In case studies, we have found many potential cancer-related lncRNAs. Most of potential association lncRNAs are supported by recent literatures. In future biological experiments, it would be interesting to find the association mechanisms between new potential lncRNAs and diseases.

**Fig. 6:**
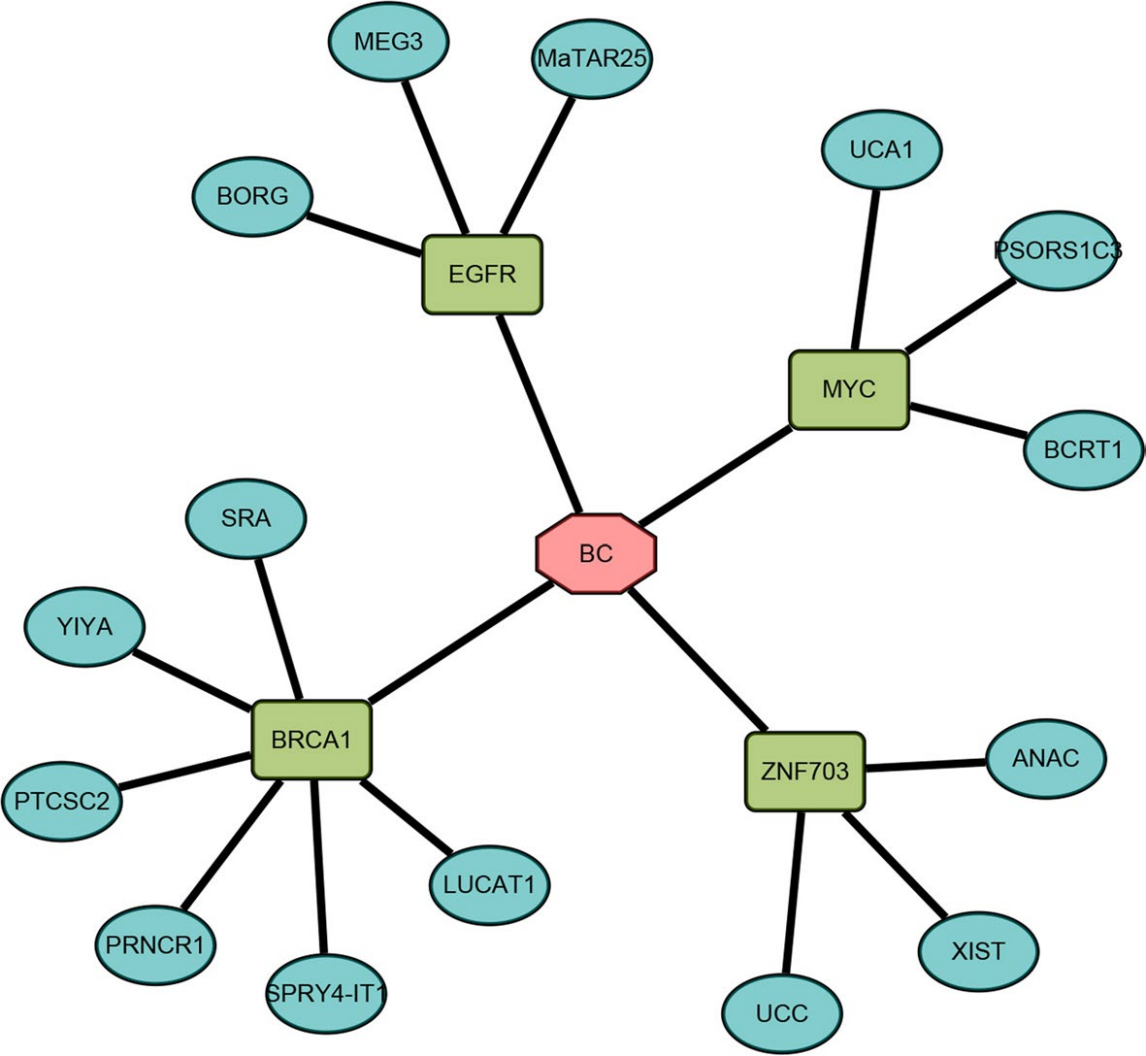
A subnet of lncRNA-gene-breast cancer (BC) network

As shown in Fig. 6, this is a sub-network discovered by our proposed method LGDLDA. The sub-network contains some confirmed lncRNAs, PSORS1C3, PTCSC2 and UCC are predicted lncRNAs not yet reported. we hypothesize the rapidly increasing biological data brings more information (e.g., Lnc2Cancer and LncACTdb), while LGDLDA combined with nonlinear mapping can more accurately capture the complex features in multi-omics data.

It should be noted that the method LGDLDA is the worst one when focusing only on top genes (FRP < 0.05 or in a lesser extent FPR < 0.1). Maybe, this is not the best method when focusing on “top prediction”. We believe that this is because the dataset is too small and affects the performance of the method. We propose two ideas to improve the performance of LGDLDA. The first idea, we use warm start strategy. We apply LGDLDA to similar training datasets to obtain a good performance parameter set β, then further optimize the parameter set β on the training set to improve the performance of LGDLDA. The second idea, we use stability selection strategy. We run LGDLDA multiple times to obtain multiple results, then use the stability selection strategy to average these results to remove the risk of overfitting caused by small datasets.

Finally, the real association mechanism between lncRNAs and disease is much more complicated than what we assumed. For example, the relationship between lncRNAs and complex diseases will change over time. We will try to design a new machine learning framework to analyze association data and time dynamic data simultaneously.

## Conclusions

In this article, we proposed a novel machine learning framework, namely LGDLDA, to find cancer-related lncRNAs by integrating analysis of multi-omics data. Firstly, LGDLDA calculates the similarity matrix of lncRNA, gene and disease respectively. LGDLDA calculates the similarity between lncRNAs through the lncRNA expression profile matrix, lncRNA-miRNA interaction matrix and lncRNA-protein interaction matrix. LGDLDA obtains gene similarity matrix by calculating the lncRNA-gene association matrix and the gene-disease association matrix. LGDLDA obtains disease similarity matrix by calculating the disease ontology, the disease-miRNA association matrix, and Gaussian interaction profile kernel similarity. Secondly, LGDLDA integrates the neighborhood information in similarity matrices by using nonlinear feature learning of neural network. Thirdly, LGDLDA uses embedded node representations to approximate the observed matrices. Finally, LGDLDA ranks candidate lncRNA-disease pairs and then selects potential disease-related lncRNAs. LGDLDA incorporates the prior knowledge of biological network topology including lncRNA similarity networks, lncRNA-gene association network, gene-disease association network, disease semantic similarity networks, and lncRNA-disease association network. In this framework, a deep learning model was used to generate feature matrices. In model optimization, the final optimization problem is a popular matrix completion problem, which can be solved using convex optimization methods. In summary, the method considers more critical information and obtains the performance improvement cancer-related lncRNA predictions.

## Methods and materials

### Overview of LGDLDA

**Fig. 7:**
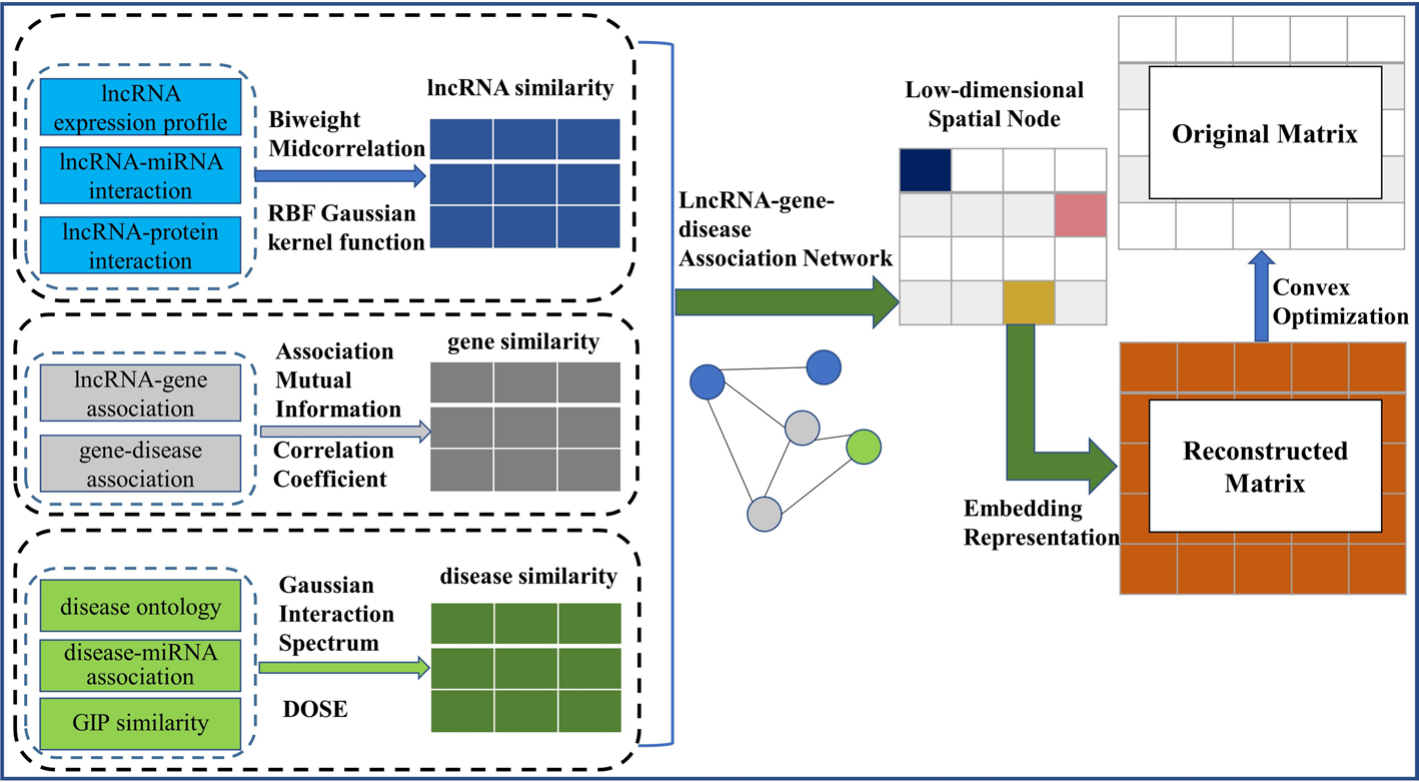
The flowchart of LGDLDA. (1) LGDLDA uses multiple association similarity matrices to build lncRNA-gene-disease association network. (2) Based on the matrices generated in the first step, LGDLDA uses the association similarity matrices combined with neural network to calculate the neighborhood information of lncRNAs and diseases, and further embeds it into the low-dimensional spatial node representations. (3) LGDLDA uses embedded representations to generate the reconstructed matrix to approximate the original matrix, and learns as much information in the original matrix as possible in the optimization of the loss function. (4) LGDLDA sorts the elements in the learned association matrix and selects the top values to predict cancer-related lncRNAs

In this section, we will introduce the main steps in the LGDLDA method. (1) LGDLDA uses multiple association similarity matrices (including lncRNA functional similarities, gene-disease associations, disease similarities, lncRNA-disease associations, and lncRNA-gene associations matrix) to build lncRNA-gene-disease association network. (2) Based on the matrices generated in the first step, LGDLDA uses the association similarity matrices combined with neural network to calculate the neighborhood information of lncRNAs and diseases, and further embeds it into the low-dimensional spatial node representations. (3) Inspired by the reconstruction matrix algorithm in NNHLDA [36], LGDLDA uses low-dimensional spatial node representations to generate the projection matrices to approximate the observed matrices, and learns as much information in the original matrix as possible in the optimization of the loss function. (4) LGDLDA sorts the elements in the learned association matrix and selects the top values to predict disease-related lncRNAs. Figure 7 shows the flowchart of LGDLDA method.

### Datasets

In this paragraph, we will introduce the mathematical formulas used next. $S \in R^{{m \times m}}$ is used to represent the lncRNAs functional similarity matrix and $D \in R^{{n \times n}}$ is used to represent disease similarity matrix, where m and n denote the number of lncRNAs and diseases, respectively. $A \in R^{{m \times n}}$ represents lncRNA-disease association matrix, rows represent lncRNAs and columns are used to represent diseases. For each entry *a*_*ij*_ in *A*, the value of *a*_*ij*_ is equal to 1 if disease *j* related to lncRNA *i*; otherwise, *a*_*ij*_ is equal to 0. Let $A_{{lg}} \in R^{{m \times k}}$ be the lncRNA-gene association matrix and $A_{{gd}} \in R^{{k \times n}}$ represents the gene-disease association matrix, where *k* represents the number of genes.

For calculating the functional similarity networks of lncRNAs, LGDLDA uses the lncRNA expression profile matrix, lncRNA-protein function association matrix and lncRNA-miRNA association matrix. For calculating the disease similarity network, LGDLDA uses disease information, protein-disease association matrix and miRNA-disease association matrix. All lncRNAs and diseases are annotated with standard corresponding IDs.

Following the work of Zhang et al. on data collection [41], LGDLDA uses the LncRNA expression data from EMBL-EBI. LncRNA-miRNA and lncRNA-protein data come from three databases including starBase v2.0 [64], NPInter v3.0 [65], and RAID v2.0 [66]. Disease-miRNA association data and disease-gene association data come from HMDD v3.0 database [67] and DisGeNet database [68] respectively. LncRNA-disease association data come from LncRNADisease v2.0 [38], Lnc2Cancer [13], and MNDR v2.0 databases [39]. Gene-lncRNA association data are collected from LncACTdb [69]. A combination of all these three datasets were used for training and validation in the article. The procedure of combination and processing are shown in the Additional file 1: Fig. S1. The combined data recruits 6000 lncRNA-disease association instances with 1724 lncRNAs and 140 diseases.

### Constructing lncRNA/disease similarity network

Since the Pearson correlation coefficient is easily affected by outliers, and outliers are inevitably included in the data, we used the biweight midcorrelation (BM) coefficient [70, 71]. Compared with Pearson correlation coefficient, the BM coefficient can calculate the correlation more accurately. We computed BM coefficients between lncRNAs and constructed the lncRNA similarity weighting network LncSm1. The range of BM value is from -1 to 1. The stronger the correlation, the larger the absolute value of BM.

The radial basis function (RBF) Gaussian kernel function was applied to lncRNA-miRNA interactions to obtain Gaussian interaction profile kernel similarity [72], and constructed the lncRNA similarity weighting network LncSm2. The similarity network can be defined as follows:

1 $$S_{{lm}} \left( {i,j} \right) = Exp\left( { - \alpha _{{l1}} \left\| {GIP_{{lm}} (l_{i} ) - GIP_{{lm}} (l_{j} )} \right\|^{2} } \right)$$

2 $$\alpha _{{l1}} = \alpha _{{l1}}^{\prime} \left( {\frac{1}{{N_{l} }}\sum\nolimits_{{i = 1}}^{{N_{l} }} {\left\| {GIP_{{lm}} (l_{i} )} \right\|^{2} } } \right)$$

where *GIP*_*lm*_(*l*_*i*_) represents the lncRNA-miRNA interaction profile, *GIP*_*lm*_(*l*_*i*_) is a binary vector in which 1 represents presence of interactions between lncRNA *l*_*i*_ and miRNA and 0 represents absence, *α*_*l*_ is the weight factor used to regulate the kernel bandwidth, the parameter $\alpha _{l}^{\prime}$ is set to 0.5 empirically and *N*_*l*_ denotes the total number of lncRNAs.

Analogous to lncRNA-miRNA interactions-based Gaussian similarity calculation method, the lncRNA-protein interactions-based Gaussian similarity of lncRNA pairs is calculated by the same method. *GIP*_*lp*_(*l*_*i*_) represents the lncRNA-protein interaction profile, *GIP*_*lp*_(*l*_*i*_) is a binary vector. With the help of the method described above, we constructed the similarity network LncSm3.

We first used the R package “DOSE” to compute the correlation coefficients between diseases [73, 74]. Then, we can build a weighted disease similarity network DisSm1. We used disease-miRNA associations to calculate the kernel similarity of the Gaussian interaction spectrum between disease *d*_*i*_ and *d*_*j*_, and then construct a weighted disease similarity correlation network DisSm2.

3 $$S_{{dm}} \left( {i,j} \right) = Exp( - \alpha _{d} \left\| {GIP_{{dm}} (d_{i} ) - GIP_{{dm}} (d_{j} )} \right\|^{2} )$$

4 $$\alpha _{d} = \alpha _{d}^{\prime} \left( {\frac{1}{{N_{d} }}\sum\nolimits_{{i = 1}}^{{N_{d} }} {\left\| {GIP_{{dm}} (d_{i} )} \right\|^{2} } } \right)$$

where *GIP*_*dm*_(*d*_*i*_) denotes disease-miRNA interaction profile, *GIP*_*dm*_(*d*_*i*_) is a binary vector.

### Constructing lncRNA/disease topological similarity networks

In order to overcome the loss of information caused by the fusion of similarity networks (i.e., LncSm1, LncSm2, and LncSm3 or DisSm1 and DisSm2), the idea of network diffusion is employed to generate the topological similarity networks. Motivated by the work of Zhang et al. [41], the RWR was applied to each similarity network to construct topological similarity network. RWR algorithm is a widely used complex biological network analysis method [41, 75, 76]. The details of constructing lncRNA/disease topological similarity networks were shown in Additional file 1. *LTS* represents the lncRNA similarity network LncTSN, and *DTS* represents the disease similarity network DisTSN.

### Node embedding

For nodes representing lncRNA or disease in the heterogeneous network, its characteristic information can be summarized from the neighbor information related to it. For example, lncRNA’s features can be aggregated from related lncRNAs, genes and diseases. Thus, we can use sufficient relevant information (related lncRNA, gene and disease information) to accurately represent the features of lncRNA. The aggregation can be defined as follows:

5 $$\begin{aligned} lnce_{i}^{\prime} & = concat\left( {lnce_{i} ,\sum\limits_{{j = 1}}^{m} {LTS^{\prime} \left\{ {i,j} \right\} \cdot \sigma _{{ll}}^{j} } } \right. \\ & \quad \left. { + \sum\limits_{{j = 1}}^{n} {A^{\prime} } \left\{ {i,j} \right\} \cdot \sigma _{{ld}}^{j} + \sum\limits_{{j = 1}}^{k} {A_{{lg}}^{\prime} \left\{ {i,j} \right\}} \cdot \sigma _{{lg}}^{j} } \right) \\ \end{aligned}$$

6 $$\begin{aligned} dise_{i}^{\prime} & = concat\left( {dise_{i} ,\sum\limits_{{j = 1}}^{n} {DTS^{\prime} \left\{ {i,j} \right\} \cdot \sigma _{{dd}}^{j} } } \right. \\ & \quad \left. { + \sum\limits_{{j = 1}}^{m} {A^{{T\prime}} } \left\{ {i,j} \right\} \cdot \sigma _{{dl}}^{j} + \sum\limits_{{j = 1}}^{k} {A_{{gd}}^{{T\prime}} \left\{ {i,j} \right\}} \cdot \sigma _{{gd}}^{j} } \right) \\ \end{aligned}$$

7 $$\begin{aligned} gee_{i}^{\prime} &= concat\left( {gee_{i} , + \sum\limits_{{j = 1}}^{m} {A_{{lg}} ^{{T\prime}} } \left\{ {i,j} \right\} \cdot \sigma _{{lg}}^{j} } \right. \\ & \quad \left. { + \sum\limits_{{j = 1}}^{n} {A_{{gd}}^{\prime} \left\{ {i,j} \right\}} \cdot \sigma _{{gd}}^{j} } \right) \\ \end{aligned}$$

where $lnce_{i}^{\prime} \in R^{{2d}}$, $dise_{i}^{\prime} \in R^{{2d}}$ and $gee_{i}^{\prime} \in R^{{2d}}$ are the embeddings of *lncRNA*_*i*_, *disease*_*i*_ and *gee*_*i*_, respectively. The initial representations of lncRNA, disease and gene nodes ($lnce_{i} \in R^{d}$, $dise_{i} \in R^{d}$ and $gee_{i} \in R^{d}$) are randomly set. By considering both node’s neighbor information and its own features, we can obtain the network topology feature information of each node, and then calculate the feature vector of this node.

The neural network obtains more powerful feature expression capability by using nonlinear activation functions. Motivated by the work of Zeng et al. [36], the activation function $\sigma \left[ \cdot \right]$ (ReLU(x) = max(x,0)) can be defined as follows:

8 $$\sigma _{{xy}}^{j} = \sigma \left( {\overline{{ye_{j} }} \cdot W_{{xy}} + b} \right)$$

where *W* and *b* denotes the parameters in the neural networks. The nodes are embedded in low-dimensional vectors and normalized:

9 $$e_{i}^{{\prime\prime}} = \frac{{\sigma \left( {e_{i}^{\prime} \cdot W_{0} + b_{0} } \right)}}{{\left\| {\sigma \left( {e_{i}^{\prime} \cdot W_{0} + b_{0} } \right)} \right\|_{2} }}$$

where $e_{i}^{{\prime\prime}}$ stands for either $lnce_{i}^{{\prime\prime}}$, $dise_{i}^{{\prime\prime}}$ or $gee_{i}^{{\prime\prime}}$. Thus, we used a single-layer neural network to non-linearly transform the nodes’ representation and obtained a new embedding representation.

### Training and evaluation

In machine learning, the model contains many parameters, and we need to use training data to determine the optimal values of the parameters through training optimization. The optimization goal is to make the difference between the predicted value and the target value (i.e., loss function) as small as possible. The information loss function between the reconstructed matrix and the original information matrix can be defined as follows:

10 $$\begin{aligned} & \mathop {\min }\limits_{{W,b,E}} \sum {\left( {A\left\{ {i,j} \right\} - lnce_{i}^{{\prime \prime }} E_{{ld1}}^{i} E_{{ld2}}^{{j^{T} }} dise_{j}^{{\prime \prime T}} } \right)^{2} } {\kern 1pt} \\ & \quad + \sum \left( {LTS\left\{ {i,j} \right\} - lnce_{i}^{{\prime \prime }} E_{{ll}}^{i} E_{{ll}}^{{jT}} lnce_{j}^{{\prime \prime T}} } \right)^{2} \\ & \quad {\kern 1pt} + \sum \left( {DTS\left\{ {i,j} \right\} - dise_{i}^{{\prime \prime }} E_{{dd}}^{i} E_{{dd}}^{{jT}} dise_{j}^{{\prime \prime T}} } \right)^{2} \\ & \quad {\text{ + }}\sum {\left( {A_{{lg}} \left\{ {i,j} \right\} - lnce_{i}^{{\prime \prime }} E_{{lg1}}^{i} E_{{lg2}}^{{jT}} gee_{j}^{{\prime \prime T}} } \right)^{2} } {\kern 1pt} \\ & \quad + \sum {\left( {A_{{gd}} \left\{ {i,j} \right\} - gee_{i}^{{\prime \prime }} E_{{gd1}}^{i} E_{{gd2}}^{{jT}} dise_{j}^{{\prime \prime T}} } \right)^{2} } \\ \end{aligned}$$

where $E \in R^{{p \times q}}$ are the information mapping matrices, which can extract the main features of the nodes from the embedded node information representations. The matrix *EE*^*T*^ is used to enforce symmetry of the recovery.

Since the functions in the method are all differentiable, we can use the gradient descent method to iteratively solve step by step to obtain the minimize loss function and model parameter values. LGDLDA uses the gradient descent method to train the model parameters. After training, elements in the reconstruction matrix can predict each associations score. The higher a score is, the larger probability we suggest the potential association exists:

11 $$A\left\{ {i,j} \right\}_{{recovered}} = lnce_{i}^{{\prime \prime }} E_{{ld1}}^{i} E_{{ld2}}^{{j^{T} }} dise_{j}^{{\prime \prime T}}$$

In this sense, the final optimization problem is a popular matrix completion problem, which can be solved using convex optimization methods.

### Evaluation method and metrics

To be able to fairly evaluate the performance of the methods, we performed LOOCV (Leave-One-Out Cross-Validation) on the verified lncRNA-disease association data. Given a disease *d*_*i*_, each known disease-related lncRNA is left out as test sample, meanwhile other disease-related lncRNAs are used as training samples. All irrelevant lncRNAs constitute candidate samples. The test samples are positive samples, and other samples are negative samples. In the predicted association matrix, LGDLDA regards elements larger than the threshold as effective associations between lncRNAs and diseases. We used true positive rate (TPR) and false positive rate (FPR) to calculate area under the curve (AUC).

## Supplementary Information

**Additional file 1: Figure S1**. The data processing procedure for disease-lncRNA association instances. **Figure S2**. The AUC values for 10 realizations on the dataset with 10% incorrect data. **Figure S3**. The box plots from 50 random splits experiment on a dataset with 10% incorrect data. **Table S1**. The experimental results on a dataset lacking some omics data. **Table S2**. The supporting literature of Top 15 gastric cancer-associated LncRNAs predicted by LGDLDA. **Table S3**. The confirmed databases of Top 15 breast cancer-associated LncRNAs predicted by LGDLDA. **Table S4**. The supporting literature of Top 15 breast cancer-associated LncRNAs predicted by LGDLDA. **Table S5**. The confirmed databases of Top 15 prostate cancer-associated LncRNAs predicted by LGDLDA. **Table S6**. The supporting literature of Top 15 prostate cancer-associated LncRNAs predicted by LGDLDA. **Table S7**. Summary of data sets used by each matrix.

## Data Availability

The software of LGDLDA is available at https://github.com/nathanyl/LGDLDA_method, to request data from this study, please contact yuanlindc@126.com. The datasets used and/or analyzed during the current study are available from the corresponding references.

## References

[1] Quinn JJ, Chang HY (2016). Unique features of long non-coding RNA biogenesis and function. Nat Rev Genet.

[2] Jarroux J, Morillon A, Pinskaya M (2017). History, discovery, and classification of lncRNAs. Long Non Coding RNA Biol.

[3] Kopp F, Mendell JT (2018). Functional classification and experimental dissection of long noncoding RNAs. Cell.

[4] Neve B, Jonckheere N, Vincent A, Van Seuningen I (2018). Epigenetic regulation by lncRNAs: an overview focused on UCA1 in colorectal cancer. Cancers.

[5] Long Y, Wang X, Youmans DT, Cech TR (2017). How do lncRNAs regulate transcription?. Science Adv.

[6] He R-Z, Luo D-X, Mo Y-Y (2019). Emerging roles of lncRNAs in the post-transcriptional regulation in cancer. Genes Dis.

[7] C.-H. Zheng, L. Yuan, W. Sha, Z.-L. Sun, Gene differential coexpression analysis based on biweight correlation and maximum clique. p. S3. 10.1186/1471-2105-15-S15-S3 PMC4271563 25474074

[8] Botti G, Collina F, Scognamiglio G, Aquino G, Cerrone M, Liguori G, Gigantino V, Malzone MG, Cantile M (2018). LncRNA HOTAIR polymorphisms association with cancer susceptibility in different tumor types. Curr Drug Targets.

[9] Peng W-X, Koirala P, Mo Y-Y (2017). LncRNA-mediated regulation of cell signaling in cancer. Oncogene.

[10] Simion V, Haemmig S, Feinberg MW (2019). LncRNAs in vascular biology and disease. Vascul Pharmacol.

[11] Zhang Y, Tang L (2018). The application of lncRNAs in cancer treatment and diagnosis. Recent Pat Anti-Cancer Drug Discovery.

[12] Chen G, Wang Z, Wang D, Qiu C, Liu M, Chen X, Zhang Q, Yan G, Cui Q (2012). LncRNADisease: a database for long-non-coding RNA-associated diseases. Nucleic Acids Res.

[13] Ning S, Zhang J, Wang P, Zhi H, Wang J, Liu Y, Gao Y, Guo M, Yue M, Wang L (2016). Lnc2Cancer: a manually curated database of experimentally supported lncRNAs associated with various human cancers. Nucleic Acids Res.

[14] Zhao Y, Li H, Fang S, Kang Y, Wu W, Hao Y, Li Z, Bu D, Sun N, Zhang MQ (2016). NONCODE 2016: an informative and valuable data source of long non-coding RNAs. Nucleic Acids Res.

[15] Amaral PP, Clark MB, Gascoigne DK, Dinger ME, Mattick JS (2011). lncRNAdb: a reference database for long noncoding RNAs. Nucleic Acids Res.

[16] Volders P-J, Helsens K, Wang X, Menten B, Martens L, Gevaert K, Vandesompele J, Mestdagh P (2013). LNCipedia: a database for annotated human lncRNA transcript sequences and structures. Nucleic Acids Res.

[17] Wang P, Ning S, Zhang Y, Li R, Ye J, Zhao Z, Zhi H, Wang T, Guo Z, Li X (2015). Identification of lncRNA-associated competing triplets reveals global patterns and prognostic markers for cancer. Nucleic Acids Res.

[18] Signal B, Gloss BS, Dinger ME (2016). Computational approaches for functional prediction and characterisation of long noncoding RNAs. Trends Genet.

[19] Wei P-J, Zhang D, Xia J, Zheng C-H (2016). LNDriver: identifying driver genes by integrating mutation and expression data based on gene-gene interaction network. BMC Bioinformatics.

[20] Chen X, Xie D, Zhao Q, You Z-H (2019). MicroRNAs and complex diseases: from experimental results to computational models. Brief Bioinform.

[21] Sun J, Shi H, Wang Z, Zhang C, Liu L, Wang L, He W, Hao D, Liu S, Zhou M (2014). Inferring novel lncRNA–disease associations based on a random walk model of a lncRNA functional similarity network. Mol BioSyst.

[22] Zhou M, Wang X, Li J, Hao D, Wang Z, Shi H, Han L, Zhou H, Sun J (2015). Prioritizing candidate disease-related long non-coding RNAs by walking on the heterogeneous lncRNA and disease network. Mol BioSyst.

[23] Zhang J, Zhang Z, Chen Z, Deng L (2017). Integrating multiple heterogeneous networks for novel lncRNA-disease association inference. IEEE/ACM Trans Comput Biol Bioinf.

[24] Yao Q, Wu L, Li J, L. guang Yang, Y. Sun, Z. Li, S. He, F. Feng, H. Li, and Y. Li,  (2017). Global prioritizing disease candidate lncRNAs via a multi-level composite network. Sci Rep.

[25] Ganegoda GU, Li M, Wang W, Feng Q (2015). Heterogeneous network model to infer human disease-long intergenic non-coding RNA associations. IEEE Trans Nanobiosci.

[26] Lu C, Yang M, Luo F, Wu F-X, Li M, Pan Y, Li Y, Wang J (2018). Prediction of lncRNA–disease associations based on inductive matrix completion. Bioinformatics.

[27] Chen X, Yan G-Y (2013). Novel human lncRNA–disease association inference based on lncRNA expression profiles. Bioinformatics.

[28] Lan W, Li M, Zhao K, Liu J, Wu F-X, Pan Y, Wang J (2017). LDAP: a web server for lncRNA-disease association prediction. Bioinformatics.

[29] Guo Z-H, You Z-H, Wang Y-B, Yi H-C, Chen Z-H (2019). A learning-based method for LncRNA-disease association identification combing similarity information and rotation forest. iScience.

[30] Engreitz JM, Haines JE, Perez EM, Munson G, Chen J, Kane M, McDonel PE, Guttman M, Lander ES (2016). Local regulation of gene expression by lncRNA promoters, transcription and splicing. Nature.

[31] Wang KC, Yang YW, Liu B, Sanyal A, Corces-Zimmerman R, Chen Y, Lajoie BR, Protacio A, Flynn RA, Gupta RA (2011). A long noncoding RNA maintains active chromatin to coordinate homeotic gene expression. Nature.

[32] Ørom UA, Derrien T, Beringer M, Gumireddy K, Gardini A, Bussotti G, Lai F, Zytnicki M, Notredame C, Huang Q (2010). Long noncoding RNAs with enhancer-like function in human cells. Cell.

[33] Wang L, Xuan Z, Zhou S, Kuang L, Pei T (2019). A novel model for predicting LncRNA-disease associations based on the LncRNA-MiRNA-Disease interactive network. Curr Bioinform.

[34] Zhao Q, Liang D, Hu H, Ren G, Liu H (2018). RWLPAP: random walk for lncRNA-protein associations prediction. Protein Pept Lett.

[35] You Z-H, Huang Z-A, Zhu Z, Yan G-Y, Li Z-W, Wen Z, Chen X (2017). PBMDA: A novel and effective path-based computational model for miRNA-disease association prediction. PLoS Comput Biol.

[36] H. Chen, X. Wang, X. Zhang, X. Zeng, T. Song, A. Rodríguez-Patón, LncRNA-disease association prediction based on neighborhood information aggregation in neural network, pp. 175–178.

[37] Yuan L, Huang D-S (2019). A network-guided association mapping approach from DNA methylation to disease. Sci Rep.

[38] Bao Z, Yang Z, Huang Z, Zhou Y, Cui Q, Dong D (2019). LncRNADisease 2.0: an updated database of long non-coding RNA-associated diseases. Nucleic Acids Res.

[39] Cui T, Zhang L, Huang Y, Yi Y, Tan P, Zhao Y, Hu Y, Xu L, Li E, Wang D (2018). MNDR v2. 0: an updated resource of ncRNA–disease associations in mammals. Nucleic Acids Res.

[40] Li G, Luo J, Liang C, Xiao Q, Ding P, Zhang Y (2019). Prediction of LncRNA-disease associations based on network consistency projection. IEEE Access.

[41] Fan X-N, Zhang S-W, Zhang S-Y, Zhu K, Lu S (2019). Prediction of lncRNA-disease associations by integrating diverse heterogeneous information sources with RWR algorithm and positive pointwise mutual information. BMC Bioinf.

[42] Wang Y, Juan L, Peng J, Zang T, Wang Y (2019). LncDisAP: a computation model for LncRNA-disease association prediction based on multiple biological datasets. BMC Bioinform.

[43] Zhang H, Liang Y, Peng C, Han S, Du W, Li Y. Predicting lncRNA-disease associations using network topological similarity based on deep mining heterogeneous networks. Math Biosci. 2019;315. 10.1016/j.mbs.2019.108229 31323239

[44] Wang J, Zhang X, Chen W, Li J, Liu C (2018). CRlncRNA: a manually curated database of cancer-related long non-coding RNAs with experimental proof of functions on clinicopathological and molecular features. BMC Med Genom.

[45] Ma L, Li A, Zou D, Xu X, Xia L, Yu J, Bajic VB, Zhang Z (2015). LncRNAWiki: harnessing community knowledge in collaborative curation of human long non-coding RNAs. Nucleic Acids Res.

[46] Network CGA (2012). Comprehensive molecular portraits of human breast tumours. Nature.

[47] L. Yuan, C.-H. Zheng, J.-F. Xia, D.-S. Huang, Module based differential coexpression analysis method for type 2 diabetes. *BioMed Res Int,* 2015, 2015. 10.1155/2015/836929 PMC4538423 26339648

[48] Fang X-Y, Pan H-F, Leng R-X, Ye D-Q (2015). Long noncoding RNAs: novel insights into gastric cancer. Cancer Lett.

[49] Yuan L, Zhu L, Guo W-L, Zhou X, Zhang Y, Huang Z, Huang D-S (2016). Nonconvex penalty based low-rank representation and sparse regression for eQTL mapping. IEEE/ACM Trans Comput Biol Bioinf.

[50] Pan L, Liang W, Fu M, Huang Z-H, Li X, Zhang W, Zhang P, Qian H, Jiang P-C, Xu W-R (2017). Exosomes-mediated transfer of long noncoding RNA ZFAS1 promotes gastric cancer progression. J Cancer Res Clin Oncol.

[51] Mao Z, Li H, Du B, Cui K, Xing Y, Zhao X, Zai S (2017). LncRNA DANCR promotes migration and invasion through suppression of lncRNA-LET in gastric cancer cells. Biosci Rep.

[52] Sun M, Nie F, Wang Y, Zhang Z, Hou J, He D, Xie M, Xu L, De W, Wang Z (2016). LncRNA HOXA11-AS promotes proliferation and invasion of gastric cancer by scaffolding the chromatin modification factors PRC2, LSD1, and DNMT1. Can Res.

[53] Liu H, Zhang Z, Wu N, Guo H, Zhang H, Fan D, Nie Y, Liu Y (2018). Integrative analysis of dysregulated lncRNA-associated ceRNA network reveals functional lncRNAs in gastric cancer. Genes.

[54] V. G. Vogel, Epidemiology of breast cancer, *The breast*, 207–218. e4. Elsevier, 2018.

[55] Ge S-G, Xia J, Sha W, Zheng C-H (2016). Cancer subtype discovery based on integrative model of multigenomic data. IEEE/ACM Trans Comput Biol Bioinf.

[56] Liang Y, Song X, Li Y, Chen B, Zhao W, Wang L, Zhang H, Liu Y, Han D, Zhang N (2020). LncRNA BCRT1 promotes breast cancer progression by targeting miR-1303/PTBP3 axis. Mol Cancer.

[57] Gooding AJ, Zhang B, Jahanbani FK, Gilmore HL, Chang JC, Valadkhan S, Schiemann WP (2017). The lncRNA BORG drives breast cancer metastasis and disease recurrence. Sci Rep.

[58] Chang K-C, Diermeier SD, Allen TY, Brine LD, Russo S, Bhatia S, Alsudani H, Kostroff K, Bhuiya T, Brogi E (2020). MaTAR25 lncRNA regulates the Tensin1 gene to impact breast cancer progression. Nat Commun.

[59] Rawla P (2019). Epidemiology of prostate cancer. World J Oncol.

[60] L. Yuan, C.-A. Yuan, D.-S. Huang, FAACOSE: A fast adaptive ant colony optimization algorithm for detecting SNP epistasis, *Complexity,* 2017, 2017.

[61] Zhao B, Lu Y-L, Yang Y, Hu L-B, Bai Y, Li R-Q, Zhang G-Y, Li J, Bi C-W, Yang L-B (2018). Overexpression of lncRNA ANRIL promoted the proliferation and migration of prostate cancer cells via regulating let-7a/TGF-β1/Smad signaling pathway. Cancer Biomark.

[62] Li J, Zhang Z, Xiong L, Guo C, Jiang T, Zeng L, Li G, Wang J (2017). SNHG1 lncRNA negatively regulates miR-199a-3p to enhance CDK7 expression and promote cell proliferation in prostate cancer. Biochem Biophys Res Commun.

[63] Zhang Y, Su X, Kong Z, Fu F, Zhang P, Wang D, Wu H, Wan X, Li Y (2017). An androgen reduced transcript of LncRNA GAS5 promoted prostate cancer proliferation. PLoS ONE.

[64] Li J-H, Liu S, Zhou H, Qu L-H, Yang J-H (2014). starBase v2. 0: decoding miRNA-ceRNA, miRNA-ncRNA and protein–RNA interaction networks from large-scale CLIP-Seq data. Nucleic Acids Res.

[65] Y. Hao, W. Wu, H. Li, J. Yuan, J. Luo, Y. Zhao, R. Chen, NPInter v3. 0: an upgraded database of noncoding RNA-associated interactions, *Database,* 2016, 2016. 10.1093/database/baw057 PMC4834207 27087310

[66] Yi Y, Zhao Y, Li C, Zhang L, Huang H, Li Y, Liu L, Hou P, Cui T, Tan P (2017). RAID v2. 0: an updated resource of RNA-associated interactions across organisms. Nucleic Acids Res.

[67] Huang Z, Shi J, Gao Y, Cui C, Zhang S, Li J, Zhou Y, Cui Q (2019). HMDD v3. 0: a database for experimentally supported human microRNA–disease associations. Nucleic Acids Res.

[68] J. Piñero, À. Bravo, N. Queralt-Rosinach, A. Gutiérrez-Sacristán, J. Deu-Pons, E. Centeno, J. García-García, F. Sanz, L. I. Furlong, DisGeNET: a comprehensive platform integrating information on human disease-associated genes and variants, *Nucleic acids research*, gkw943, 2016. 10.1093/nar/gkw943 PMC5210640 27924018

[69] Wang P, Li X, Gao Y, Guo Q, Wang Y, Fang Y, Ma X, Zhi H, Zhou D, Shen W (2019). LncACTdb 2.0: an updated database of experimentally supported ceRNA interactions curated from low-and high-throughput experiments. Nucleic Acids Res.

[70] Langfelder P, Horvath S (2012). Fast R functions for robust correlations and hierarchical clustering. J Stat Softw.

[71] Yuan L, Guo L-H, Yuan C-A, Zhang Y, Han K, Nandi AK, Honig B, Huang D-S (2018). Integration of multi-omics data for gene regulatory network inference and application to breast cancer. IEEE/ACM Trans Comput Biol Bioinf.

[72] van Laarhoven T, Nabuurs SB, Marchiori E (2011). Gaussian interaction profile kernels for predicting drug–target interaction. Bioinformatics.

[73] Yu G, Wang L-G, Yan G-R, He Q-Y (2015). DOSE: an R/Bioconductor package for disease ontology semantic and enrichment analysis. Bioinformatics.

[74] Li J, Gong B, Chen X, Liu T, Wu C, Zhang F, Li C, Li X, Rao S, Li X (2011). DOSim: an R package for similarity between diseases based on disease ontology. BMC Bioinform.

[75] Luo Y, Zhao X, Zhou J, Yang J, Zhang Y, Kuang W, Peng J, Chen L, Zeng J (2017). A network integration approach for drug-target interaction prediction and computational drug repositioning from heterogeneous information. Nat Commun.

[76] Gligorijević V, Barot M, Bonneau R (2018). deepNF: deep network fusion for protein function prediction. Bioinformatics.

