# Supplementary material for: A machine learning framework that integrates multi-omics data predicts cancer-related LncRNAs
Source: BMC Bioinformatics. 2021 Jun 16;22:332. doi: 10.1186/s12859-021-04256-8 (PMC8210375; doi:10.1186/s12859-021-04256-8)
Supplement: Supplementary file 1 — Additional file 1: Figure S1. The data processing procedure for disease-lncRNA association instances. Figure S2. The AUC values for 10 realizations on the dataset with 10% incorrect data. Figure S3. The box plots from 50 random splits experiment on a dataset with 10% incorrect data. Table S1. The experimental results on a dataset lacking some omics data. Table S2. The supporting literature of Top 15 gastric cancer-associated LncRNAs predicted by LGDLDA. Table S3. The confirmed databases of Top 15 breast cancer-associated LncRNAs predicted by LGDLDA. Table S4. The supporting literature of Top 15 breast cancer-associated LncRNAs predicted by LGDLDA. Table S5. The confirmed databases of Top 15 prostate cancer-associated LncRNAs predicted by LGDLDA. Table S6. The supporting literature of Top 15 prostate cancer-associated LncRNAs predicted by LGDLDA. Table S7. Summary of data sets used by each matrix. [file 12859_2021_4256_MOESM1_ESM.pdf]

# A Machine Learning Framework that Integrates Multi-omics Data Predicts Cancer-related LncRNAs

## ——Supplementary Materials

Lin Yuan[1], Tao Sun[1], Jing Zhao[1], and Zhen Shen[2*]

## 1. Figure S1

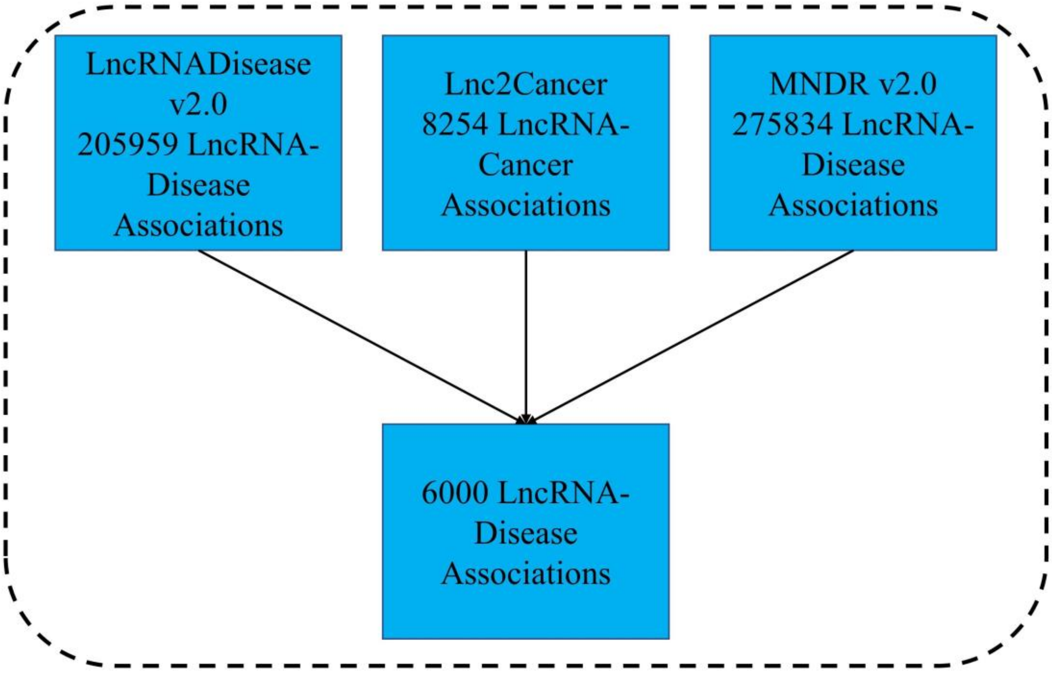


**Fig. S1.** The data processing procedure for disease-lncRNA association instances. (1) We only kept lncRNA-disease associations from Homo sapiens species. (2) We removed some associations due to the availability of multiomic data. (3) We kept some associations involving with gastric, breast and prostate cancer for later use.

• Correspondence: wfxueyuan@126.com
[1]School of Computer Science and Technology, Qilu University of Technology (Shandong Academy of Sciences), Daxue Road 3501, Jinan, Shandong 250353, China.
[2]School of Computer and Software, Nanyang Institute of Technology, Changjiang Road 80, Nanyang, Henan 473004, China.
Full list of author information is available at the end of the article.

## 2. Figure S2

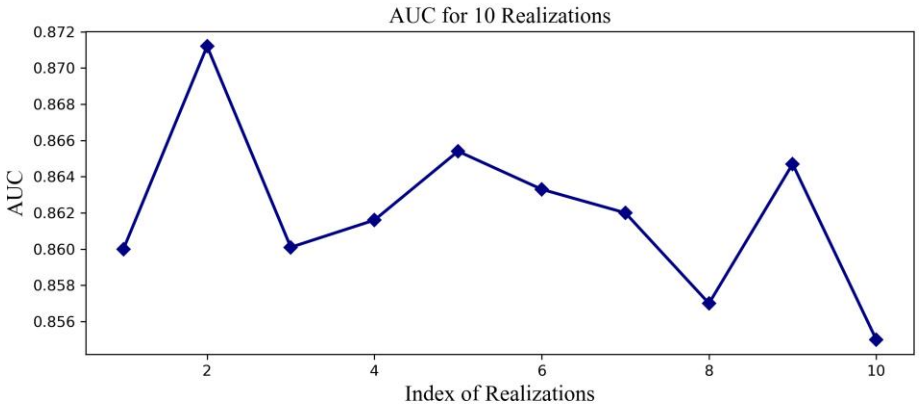


**Fig. S2.** The AUC values for 10 realizations on the dataset with 10% incorrect data.

## 3. Figure S3

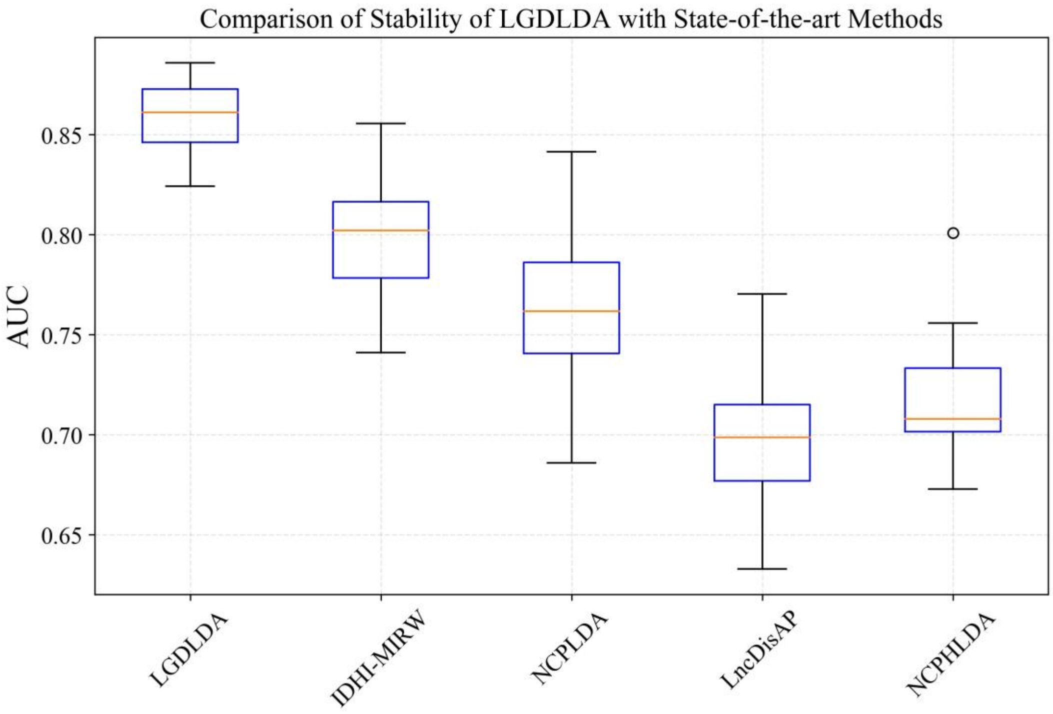


**Fig. S3.** The box plots from 50 random splits experiment on a dataset with 10% incorrect data.

## 4. Table S1

**Table S1** The experimental results on a dataset lacking some omics data.

| Dataset / AUC Values | Multi-omics Information | Multi-omics Information (Missing Gene Information) | Multi-omics Information (Missing LncRNA-Gene Information) | Multi-omics Information (Missing Gene-Disease Information) | Multi-omics Information (Missing LncRNA-Gene Information) |
|---|---|---|---|---|---|
| AUC-1 | 0.912 | 0.820 | 0.745 | 0.779 | 0.662 |
| AUC-2 | 0.910 | 0.790 | 0.701 | 0.774 | 0.625 |
| AUC-3 | 0.914 | 0.819 | 0.729 | 0.773 | 0.637 |
| AUC-4 | 0.906 | 0.869 | 0.699 | 0.72 | 0.664 |
| AUC-5 | 0.910 | 0.865 | 0.725 | 0.765 | 0.574 |
| AUC-6 | 0.915 | 0.847 | 0.694 | 0.762 | 0.684 |
| AUC-7 | 0.911 | 0.853 | 0.728 | 0.778 | 0.630 |
| AUC-8 | 0.913 | 0.828 | 0.771 | 0.729 | 0.650 |
| AUC-9 | 0.908 | 0.813 | 0.729 | 0.75 | 0.645 |
| AUC-10 | 0.907 | 0.875 | 0.687 | 0.708 | 0.623 |
| Average | 0.9106 | 0.8379 | 0.7208 | 0.7538 | 0.6394 |

## 5. Table S2

**Table S2** The supporting literature of Top 15 gastric cancer-associated LncRNAs predicted by LGDLDA.

| Rank | Name of LncRNA | Verified | Literatures (PMID) |
|---|---|---|---|
| 1 | UCA1 | Yes | 28075173 |
| 2 | NEHG1 | No | without evidence |
| 3 | ZFAS1 | Yes | 28285404 |
| 4 | HOTAIR | Yes | 24949306 |
| 5 | C1RL-AS1 | No | without evidence |
| 6 | H19 | Yes | 24810858 |
| 7 | PVT1 | Yes | 27756785 |
| 8 | NEAT1 | Yes | 29363783 |
| 9 | MEG3 | Yes | 26253106 |
| 10 | MALAT1 | Yes | 28268166 |
| 11 | DM1-AS | No | without evidence |
| 12 | DANCR | Yes | 28951520 |
| 13 | GHET1 | Yes | 24397586 |
| 14 | FER1L4 | Yes | 24961353 |
| 15 | HOXA11-AS | Yes | 27651312 |

## 6. Table S3

**Table S3** The confirmed databases of Top 15 breast cancer-associated LncRNAs predicted by LGDLDA.

| Rank | Name of LncRNA | Confirmed Database |
|---|---|---|
| 1 | BCRT1 | CRlncRNA |
| 2 | BORG | CRlncRNA/Lnc2Cancer/LncRNAWiki/LncRNADisease v2.0 |
| 3 | MaTAR25 | CRlncRNA |
| 4 | SPRY4-IT1 | MNDRv2.0/Lnc2Cancer/LncRNADisease v2.0 |
| 5 | PSORS1C3 | Unconfirmed |
| 6 | MEG3 | LncRNAWiki/Lnc2Cancer/LncRNADisease v2.0 |
| 7 | PRNCR1 | CRlncRNA/Lnc2Cancer |
| 8 | UCA1 | LncRNADisease v2.0/Lnc2Cancer |
| 9 | PTCSC2 | Unconfirmed |
| 10 | ANAC | Lnc2Cancer/CRlncRNA |
| 11 | UCC | Unconfirmed |
| 12 | SRA | CRlncRNA/LncRNADisease v2.0/Lnc2Cancer |
| 13 | XIST | Lnc2Cancer/LncRNADisease v2.0/CRlncRNA |
| 14 | YIYA | Lnc2Cancer/LncRNAWiki/LncRNADisease v2.0 |
| 15 | LUCAT1 | CRlncRNA/Lnc2Cancer |

## 7. Table S4

**Table S4** The supporting literature of Top 15 breast cancer-associated LncRNAs predicted by LGDLDA.

| Rank | Name of LncRNA | Verified | Literatures (PMID) |
|---|---|---|---|
| 1 | BCRT1 | Yes | 31300015 |
| 2 | BORG | Yes | 28983112 |
| 3 | MaTAR25 | Yes | 33353933 |
| 4 | SPRY4-IT1 | Yes | 31736268 |
| 5 | PSORS1C3 | No | without evidence |
| 6 | MEG3 | Yes | 30793226 |
| 7 | PRNCR1 | Yes | 31798697 |
| 8 | UCA1 | Yes | 31695578 |
| 9 | PTCSC2 | No | without evidence |
| 10 | ANAC | Yes | 29795261 |
| 11 | UCC | No | without evidence |
| 12 | SRA | Yes | 30238005 |
| 13 | XIST | Yes | 30026327 |
| 14 | YIYA | Yes | 29967256 |
| 15 | LUCAT1 | Yes | 31300015 |

## 8. Table S5

**Table S5** The confirmed databases of Top 15 prostate cancer-associated LncRNAs predicted by LGDLDA.

| Rank | Name of LncRNA | Confirmed Database |
|---|---|---|
| 1 | PCA3 | CRlncRNA/Lnc2Cancer/LncRNAWiki/LncRNADisease v2.0 |
| 2 | TTTY15 | CRlncRNA/Lnc2Cancer |
| 3 | HCP5 | CRlncRNA/Lnc2Cancer |
| 4 | CCAT2 | Lnc2Cancer/LncRNADisease v2.0 |
| 5 | GAS5 | CRlncRNA/Lnc2Cancer/LncRNADisease v2.0 |
| 6 | MSC-AS1 | Unconfirmed |
| 7 | LINP1 | CRlncRNA/Lnc2Cancer |
| 8 | TUG1 | CRlncRNA/Lnc2Cancer |
| 9 | ZFAS1 | CRlncRNA/Lnc2Cancer |
| 10 | SLINKY | Unconfirmed |
| 11 | RNCR3 | CRlncRNA/Lnc2Cancer |
| 12 | GLIDR | CRlncRNA/Lnc2Cancer |
| 13 | PCSEAT | CRlncRNA/Lnc2Cancer/LncRNAWiki |
| 14 | IRAIN | Unconfirmed |
| 15 | PCOTH | CRlncRNA/Lnc2Cancer/LncRNAWiki |

## 9. Table S6

**Table S6** The supporting literature of Top 15 prostate cancer-associated LncRNAs predicted by LGDLDA.

| Rank | Name of LncRNA | Verified | Literatures (PMID) |
|---|---|---|---|
| 1 | PCA3 | Yes | 30016891 |
| 2 | TTTY15 | Yes | 30527798 |
| 3 | HCP5 | Yes | 31746434 |
| 4 | CCAT2 | Yes | 32831916 |
| 5 | GAS5 | Yes | 31673232 |
| 6 | MSC-AS1 | No | without evidence |
| 7 | LINP1 | Yes | 30058678 |
| 8 | TUG1 | Yes | 30915735 |
| 9 | ZFAS1 | Yes | 32104094 |
| 10 | SLINKY | No | without evidence |
| 11 | RNCR3 | Yes | RNCR3 |
| 12 | GLIDR | Yes | 28211799 |
| 13 | PCSEAT | Yes | 29803673 |
| 14 | IRAIN | No | without evidence |
| 15 | PCOTH | Yes | 15930275 |

## 10. Table S7

**Table S7** Summary of data sets used by each matrix.

| Matrix | Dataset | | |
|---|---|---|---|
| LncSm1 | EMBL-EBI | | |
| LncSm2 | starBase v2.0 | NPInter v3.0 | RAID v2.0 |
| LncSm3 | starBase v2.0 | NPInter v3.0 | RAID v2.0 |
| Gene Similarity Matrix | DisGeNet | LncACTdb | |
| DisSM1 | LncRNADisease v2.0 | Lnc2Cancer | MNDR v2.0 |
| DisSM2 | HMDD v3.0 | | |

## 11. The details of "*Constructing lncRNA/disease topological similarity networks*"

The RWR can be defined as follows:

$$M^{t+1} = (1-\alpha)M^t W + \alpha M^0 \quad (1)$$

$$W(i,j) = \frac{B(i,j)}{\sum_j B(i,j)} \quad (2)$$

where $M^t$ represents the distribution probability matrix of one node visiting another node, and $M^0$ denotes the initial access probability distribution matrix. $\alpha$ represents the probability of restart. For lncRNA or disease, $B$ denotes the edge-weighted adjacency matrix.

$$TS(i,j) = max(0, \log_2 \frac{M(i,j)\sum_i \sum_j M(i,j)}{\sum_i M(i,j) \sum_j M(i,j)}) \quad (3)$$

where matrix $TS$ is an asymmetric matrix, in which we use the value $(TS(i,j)+TS(j,i))/2$ to represent the similarity value of the network topology between node $i$ and node $j$. The values in the integration lncRNA network topological similarity matrix $LTS$ can be obtained by calculating the average values of the corresponding position elements in the three lncRNA topological similarity matrices $LTS_1$, $LTS_2$, $LTS_3$ of LncSm1, LncSm2 and LncSm3. The values in the disease network topological similarity matrix DTS can be obtained by calculating the average values of the corresponding position elements in the two disease network topological similarity matrices $DTS_1$, $DTS_2$ of DisSm1, DisSm2.
